# Measuring the productivity of residential long-term care in England: methods for quality adjustment and regional comparison

**DOI:** 10.1007/s10198-016-0816-z

**Published:** 2016-07-16

**Authors:** Wei Yang, Julien Forder, Olena Nizalova

**Affiliations:** Personal Social Services Research Unit, George Allen Wing, University of Kent, Kent, Canterbury, CT2 7NF UK

**Keywords:** Regional productivity, Care home, England, I10

## Abstract

Productivity trend information is valuable in developing policy and for understanding changes in the ‘value for money’ of the care system. In this paper, we consider approaches to measuring productivity of adult social care (ASC), and particularly care home services. Productivity growth in the public sector is traditionally measured by comparing change in total output to change in total inputs, but has not accounted for changes in service quality and need. In this study, we propose a method to estimate ‘quality adjusted’ output based on indicators of the Adult Social Care Outcomes Toolkit (ASCOT), using data collected in the annual adult social care survey (ASCS). When combined with expenditure and activity data for 2010 to 2012, we found that this approach was feasible to implement with current data and that it altered the productivity results compared with non-adjusted productivity metrics. Overall, quality-adjusted productivity grew in most regions between 2010 and 2011 and remained unchanged for most regions from 2011 to 2012.

## Introduction

Demographic change and financial pressures are combining to create a challenging environment for adult long-term (social) care in England and elsewhere. In this context, there has been greater attention to issues of productivity and value for money [1]. Nonetheless, measures of productivity in this field have so far been limited and potentially misleading, particularly by failing to account for the quality of the care system, not just the amount of output it produces. The aim of this paper is to propose a novel approach to productivity measurement in long-term care that adjusts for patient outcomes, and so provides a more accurate picture for policy-makers. The paper also provides (quality-adjusted) productivity comparisons between regions in England.

Almost all public-funded adult social care in England is organized through local authorities (LAs) [2, 3]. With regard to ASC responsibilities, LAs operate with a framework of legislation and guidance from the government. In line with the principles consolidated in recent legislation (the 2014 Care Act), the aim of the system is to improve the well-being of the population with care needs. In this way, the care system is assessed within the Adult Social Care Outcomes Framework (ASCOF) implemented by the Department of Health (DH) [4]. As such, LAs are assessed on their achievements—improved care-related outcomes for their local population, as measured in the ASCOF—while working within a given funding envelope.

In the UK, there is a growing body of research on public service productivity. The Office of National Statistics (ONS) provided two key reports on productivity in ASC services in 2006 and 2007 [5, 6]. A range of data on inputs and outputs were used to construct national productivity trends for adult social care (ASC) services between 1996 and 2005. However, the reports also acknowledged a number of limitations. First, those measures of productivity ought to include an output index, which incorporates quality change. Second, the output measures used were based largely upon numbers of people receiving services and did not account for the consequences of any changes in the average level of need of clients. Existing evidence suggests that the average level of need of older people in care homes has increased by 10–16 % between 1995 and 2005, approximately 1 % per year [7]. Therefore, the measures provided by the ONS are considered as basic (unadjusted) ‘productivity’ estimates that only compare change in costs with changes in levels of activity.

Compared to adult social care, there is more research on productivity in the healthcare sector in England [8–12]. A number of relevant critical issues were taken into account in these studies. The methods used were able to capture a range of health services delivered to NHS patients; they make use of routine collection of health outcome data to adjust for quality of output; and are capable of being disaggregated both to different settings and to sub-national levels.

As the purpose of the care system is to improve (care-related) quality of life in the population of people with care needs, the ‘output’ of the system should ideally be measured by the change in quality of life that it produced. A pragmatic approach is to measure activity but then to apply an adjustment to reflect (change in) the contribution of local activity to quality of life [13]. This adjustment would capture the ‘quality’ of care locally in terms of how well it improved care-related quality of life in the local population. We propose to compare changes in care-related quality of life in the population of people using care services in each locality. The cross-sectional annual Adult Social Care Survey (ASCS) provides such data and is sufficiently large to give a reasonable indication of population care-related quality of life at the local authority level. This approach does not differentiate between the different services people use. Rather, quality adjustment involves weighting total output for changes over time in the care-related quality of life of the service user population. This approach accounts for need by netting out changes in care-related quality of life due to changes in individual needs-related factors measured in the survey. Conceptually, ‘need’ can be regard as the person’s quality of life without services. For example, people with high need would have low quality of life without services compared to people with low need. Productivity is measured in terms of the *improvement* in quality of life produced by services and is the difference between current quality of life and quality of life without services—that is ‘need’. Our approach estimates this difference using data on current quality of life and on need factors.

As well as assessing the difference made by our approach to quality adjustment, the resulting estimates of productivity can be used to compare care systems geographically and through time. We concentrate on the case of care home services (residential care) for older people and measure productivity growth from 2010 to 2012 across regions in England. At present, historic data from the Adult Social Care Survey is limited, but as new survey data becomes available, the time-trend comparisons can be extended.

The next section provides a review of productivity measurements and justification of the study framework, followed by methods, results, discussion, and conclusions.

## Quality measurements in productivity analysis in the social care sector in England

The National Accounts first introduced the methodology used to measure ASC productivity following implementation of the recommendations of the Atkinson Review in 2005 [8]. The methodology involved measuring the level of social services activities, either in terms of time (e.g., number of weeks of residential care) or number of items (e.g., number of meals provided). The activities covered a range of services: professional advice and support, residential and nursing care, day care, meals, home care, etc. Services were measured separately for different client groups: people over 65 and younger adults with disabilities or other health needs.

Productivity is conventionally defined as the ratio of output to input. For the market sector of the economy, the numerator is constructed by aggregating the volume of goods and services using prices as weights, the assumption being that prices reflect the consumer’s marginal willingness to pay and, hence, marginal social welfare [14]. However, for the majority of public goods, there are no prices to indicate the relative values of these goods. In the absence of information on prices or other information about the marginal contribution to welfare of each ASC service, the default approach in the National Accounts has been to use unit costs to reflect relative values, albeit with quality adjustment where possible [15].

Data on costs are readily available, but this information cannot be assumed to reflect consumer valuation. Quality adjustment is argued to improve measurement of the value of outputs, although a number of conditions would apply [5, 13, 15].

Studies to date have suggested three aspects of quality indicators for ASC—structure, process, and outcomes [16, 17]. Structure quality indicators usually refer to the ‘relatively stable characteristics of the providers of care, of the tools and resources they have at their disposal, and of the physical and organizable settings in which they work’ [18]. In ASC, relevant indicators would be whether care homes offer single-occupancy rooms, the size of rooms, and the range of facilities available in a care home, etc. However, the problem of using these characteristics as quality indicators is that they are relatively insensitive to changes over time, and will not be sufficient to measure quality. They lack the core focus of quality assessment—the carer-service user relationship. In areas where the services do not have physical attributes, for example, where the carer provide services such as dressing, feeding, or their attitudes towards service users, it is difficult to identify relevant indicators. Although some data on the qualifications and employment experience of these carers could be used, these characteristics are often considered as poor predictors of quality [16, 17].

A relevant outcome approach in ASC is to use preference-weighted social care-related quality of life (SCRQoL) measures to rate the valued consequences of care services [13, 21, 22]. This approach allows for different forms of ASC to be compared in the same quality-of-life ‘currency’, where the value of the care service is rated on a scale anchored between full care-related quality of life (1) and a quality of life equivalent to death (0). The ‘care-related quality of life’ of people with care needs has close conceptual resonance with the idea of these people’s well-being.

The Adult Social Care Outcomes Toolkit (ASCOT) includes a number of SCRQoL measures. ASCOT was developed to measure social care outcome and process in eight conceptually distinct attributes: personal cleanliness and comfort, food and drink, control over daily life, personal safety, accommodation cleanliness and comfort, social participation and involvement, occupation, and dignity [22]. Among these eight domains, dignity is included to reflect the impacts of the care process on how people feel about themselves. ASCOT has been cognitively tested and demonstrates good psychometric properties [13, 16, 17, 23], relevance, and sensitivity [21]. The main ASCOT measure is a core indicator in the Adult Social Care Outcomes Framework (ASCOF).

Process indicators of quality in social care generally measure the way in which care is delivered. For example, by asking whether carers devote enough time to care tasks, whether there are good relationships between the service user and care staff, and so on. These are likely to be important predictors of final outcomes for people using services, but this approach requires that we assume that good process means good outcomes. Also, as noted, tools like ASCOT do account for aspects of process in terms of the impact this has on people’s sense of dignity. Ultimately, we argue that outcome indicators, as direct indicators of the final impact of the services, are preferable for the purpose of comparing quality across different types of services. This position is endorsed in policy with ministers stating that the objective of services is to provide ‘better outcomes for all’ [19, 20].

Measuring service quality requires being able to remove possible contributions to outcomes of services and non-service factors, which are often referred to as an ‘attribution problem’ [24–26].

Two specific types of attribution problem are commonly noted in the literature. The first is in relation to clients’ needs. Service use is found to be positively related to care needs, and negatively related to care-related quality of life (ASCOT), because people with higher levels of need tend to require more support but, other things equal, will show worse quality of life. Taking an outcomes perspective, need can be thought of as a deficit in quality of life, that is, the quality of life of an individual in the absence of services. Assuming two service users have the same current ASCOT score, a week of care in a care home delivered to the client with less severe disability cannot be considered the same as a week of caring for the client with high disability. The client with high disability must have received relatively better *quality* *of care services* in order to produce the same level of social care-related quality of life as the one with less severe disability. Since our aim is to measure changes in quality of care services, it is important to control for the direct effect of need on SCRQoL. Need in the population will vary over time, but we would want to avoid falsely attributing changes in SCRQoL to changes in the quality of care services if that change was actually related to changes in need.

The second attribution problem is to understand factors that are beyond service. There are a range of external factors that will affect the current ASCOT of service users, as well as the impact of the care system [16]. A number of researchers have started identifying potential non-service-related factors affecting social care outcome. Fernandez et al. [23] found that ASC service coverage was lower than the observed one after controlling for regional demographic and socioeconomic characteristics. These findings also suggested a need to adjust regional non-service factors that are likely to bias the assessment of local performance.

Attribution problems can be addressed in a number of ways. The conventional approach is to use randomized control trials (RCTs) or similar experimental methods. Observational or non-experimental methods that are suitable for productivity analyses involve the use of statistical models to control for other, non-service, factors that affect ASCOT [27].

Turning to the denominator of the productivity ratio, it is necessary to measure changes in input. Two different methods of measuring input growth have been studied: direct and indirect measures (deflated expenditure measure). Input is usually categorized into three broad categories: labor (e.g., administrative, professional, technical and clerical, social workers, occupational therapists), intermediate inputs (e.g., procurement) and capital inputs (e.g., buildings and equipment). The direct measure of input is the product of volume and price of direct input [9, 10]. The indirect measure is the expenditure incurred in the direct provision of care. For example, in care homes this includes expenditures on food, utilities, and the provision of other items necessary for daily living. It is important to remove the effects of price inflation from expenditure data, using a suitable deflator.

In the case of ASC, direct measurement can rarely be undertaken because comprehensive information on the amount of inputs is seldom available. Information on expenditure is available—from annual financial reports that LAs provide to the Department of Health (DH) (the PSS-EX1 return). In this study, Pay and Prices Index is used as a deflator.

## Methods

### Measuring output in ASC

We measure output in terms of time spent on residential and nursing care activities (i.e., number of weeks of residential or nursing care) for older people over 65. A *cost-weighted output index* is constructed as the percentage change in volume of each output weighted by the cost of each service (*k*) (in this case, *k* = residential and nursing care for older people). Therefore, in a Laspeyres form, output growth for each LA (*i*) for residential and nursing care services for older people is written as [10, 12, 15]:

1 $$I_{it + 1} = \frac{{\sum\limits_{k} {x_{kit + 1} c_{kit} } }}{{\sum\limits_{k} {x_{kit} c_{kit} } }}$$

where $I_{it + 1}$ is the *output growth index*, which is a function of *x*_*kit*_, the volume of residential and nursing care service for older people in period *t*, and *c*_*kit*_ is the unit cost of the service output.

### Quality adjustment using individual level data

We use data from the Adult Social Care Survey (ASCS) as the basis for quality adjustment [8, 11, 12, 16, 28]. The ASCOT score is calculated using time trade-off (TTO) method [22]. The score has a range from 0 to 1, with ‘0’ equivalent to ‘being dead’ and ‘1’ being the ‘ideal’ SCRQoL state. Following discussion in "Quality measurements in productivity analysis in the social care sector in England", we assume that the individual person ASCOT score *y*_*jit*_ is a function of individual’s needs *σ*_*jit*_, demographic characteristics *θ*_*jit*_ and the amount of care the person receives—the vector of *k* services, $x_{jit}^{k}$. Since almost all public-funded adult social care in England is organized through local authorities (LAs) [2, 3], the influence of service quality on people’s care-related quality of life (ASCPT) will be correlated at the LA-level, but this effect cannot be directly observed in the data. Rather, we use an unobserved ‘quality of care’ factor *q*_*it*_. Here, the subscript *j* denotes the individual person, within LA *i* at time *t*. The unobserved quality of care in the area consists of two components: time constant $\tilde{q}_{i}$ and time-varying $\tilde{q}_{it}$. ASCOT is therefore:

2 $$y_{jit} = y_{jit} (\tilde{q}_{i} ,\tilde{q}_{it} ,\tilde{r}_{i } , \tilde{r}_{it} , \sigma_{jit} ,\theta_{jit} ,x_{jit}^{k} ).$$

It would be ideal to capture as far as possible LA level characteristics that may influence ASCOT, denoted *r*_*it*_. These LA-level variables can be time invariant, $\tilde{r}_{i}$, and time-varying, $\tilde{r}_{i}$.

We specify the following individual level regression model with LA-specific fixed effects:

3 $$y_{jit} = \alpha_{it} \left( {q_{it} , r_{it} } \right) + z_{jit}^{\sigma } \beta_{1} + z_{jit}^{\theta } \beta_{2} + \varepsilon_{jit}$$

where the *z* terms are the available individual level proxies for need and demographics, respectively.

In this model, *ε*_*jit*_ is the idiosyncratic error term, which will reflect missing factors. We do not have data on individual person service use, and we only observe a subset of need and other factors. Unobserved effects will therefore show in the error. In this regard, it is useful to think of the error having two components: $\varepsilon_{jit} = f^{x} \left( {\left. {x_{jit}^{k} ,\sigma_{jit} ,\theta_{jit} } \right|z_{jit}^{\sigma } ,z_{jit}^{\theta } ,\tilde{q}_{i} ,\tilde{q}_{it} ,\tilde{r}_{it } , \tilde{r}_{i} } \right) + \epsilon_{jit} (\sigma_{jit} ,\theta_{jit} )$ where $f^{x} (.)$ is the impact of services on ASCOT (but with effects that are in addition to LA-level service quality and observed needs, which are captured directly in the equation). The choice of the model is determined by the nature of the question as we are interested in the estimates of all LA-specific time effects.

The term $\alpha_{it} (q_{it} , r_{it} )$ is our quality of care adjustment, and with reference to (3) measures the change over time in quality of life (i.e., Δ*y*_*it*,*t*−1_), controlling for changes in need. If person-level quality of life *y*_*jit*_ increased on average, for example, and other factors such as individual need, service intensity, etc., stayed the same, we would conclude that quality had increased: Δ*y*_*it*,*t*−1_ would be bigger. Alternatively, if a change in person-level quality of life was due *entirely* to the opposite change in need, then $\alpha_{it} (q_{it} , r_{it} )$, would not increase. Services in this case would not have become more productive, just dealing with a different case-mix; they would be improving quality of life by the same degree. However, if need increased and current quality of life remained unchanged, then $\alpha_{it} (q_{it} , r_{it} )$ (and so Δ*y*_*it*,*t*−1_) would also increase, since services would be increasing quality of life by a greater degree—the ‘before-services’ quality of life would be lower if need was higher. This would be an increase in productivity, which would, in theory, be captured by this method.

With reference to (3), *α*_*it*_ is the LA level-time effect on ASCOT, which consists of year variables, LA variables, and interaction terms of these two sets of variables. The LA level-time effect in the model will capture quality effects but also a subset of any missing need and supply factors, which are invariant at the individual person level. Assuming a linear association, we have: $\alpha_{it} = \alpha_{1} \left( {\tilde{q}_{it} + \tilde{q}_{i} } \right) + \alpha_{2} (\tilde{r}_{it} + \tilde{r}_{i } )$, where $\tilde{r}_{i}$ and $\tilde{r}_{it}$ are other LA-level need/supply effects.

In order to obtain the year quality change ratio, we are interested in $\frac{{\tilde{q}_{it + 1} + \tilde{q}_{i} }}{{\tilde{q}_{it} + \tilde{q}_{i} }}$. If we assume that the other individual level invariant effects and the constant are small, i.e., $\alpha_{2} \cong 0$,^1^ then the change in the year-to-year quality of care is:

4 $$\frac{{\hat{Q}_{it + 1} }}{{\hat{Q}_{it} }} = \frac{{\tilde{q}_{it + 1} + \tilde{q}_{i} }}{{\tilde{q}_{it} + \tilde{q}_{i} }} \cong \frac{{\alpha_{it + 1} }}{{\alpha_{it} }}.$$

Since need and other demographic variables tend to vary at the individual person level, this supports our assumption that $\tilde{r}_{i }$ and $\tilde{r}_{it}$ are small. Local supply factors might be individual level invariant, but there is some debate as to whether they might be regarded as quality factors anyway.

Through the assessment process, the care system determines $x_{jit}^{k}$ as a function of need and other factors, including the terms $z_{jit}^{\sigma }$ and $z_{jit}^{\theta }$ in (3). However, because this relationship could differ from the relationship between observed need and current ASCOT for the individual, there is a potential endogeneity problem in estimating (3).

In the estimation, some of the effects of services will be captured in the need variables. In turn, we might expect some bias in the estimation of *α*_*it*_, although again the effect on the ratio $\alpha_{it + 1} /\alpha_{it}$ should be small because there is no reason to believe that the bias is time-invariant. This effect should be noted, but should be considered against the alternatives of either making a quality adjustment with the crude ratio $\bar{y}_{it + 1} /\bar{y}_{it}$ (where $\bar{y}_{it}$ is the LA-mean value of ASCOT), or making no adjustment.

Since our approach involves estimating descriptive LA-level statistics on the basis of sample data, we apply sample weights in the analysis of quality adjustment. Equation (4) will be used to estimate a cost-weighted quality adjusted *output index*:

5 $$I_{it + 1}^{Q} = \frac{{\sum\limits_{k} {x_{kit + 1} c_{kit} } }}{{\sum\limits_{k} {x_{kit} c_{kit} } }}\frac{{\hat{Q}_{it + 1} }}{{\hat{Q}_{it} }} = \frac{{\sum\limits_{k} {x_{kit + 1} c_{kit} } }}{{\sum\limits_{k} {x_{kit} c_{kit} } }}\frac{{\alpha_{it + 1} }}{{\alpha_{it} }}.$$

### Measuring input and productivity in ASC

Drawing from the discussion in "Quality measurements in productivity analysis in the social care sector in England", the total input of social care can be measured by the money spent on adult social care by the social services department in LAs in England, and this should be equivalent to the product of volume and price of direct input. We use an *indirect input growth index*:

6 $$Z_{it + 1} = \frac{{\sum\limits_{g = 1}^{G} {\delta_{gt} E_{gt + 1} } }}{{\sum\limits_{g = 1}^{G} {E_{t} } }}$$

where *E*_*g*_ is expenditure on input type *g*. A deflator *δ*_*gt*_ is applied to input *g* to wash out the effect of price rises in expenditure growth [9].

Using the output and input indices, the overall *productivity growth index* [10] for ASC is:

7 $$P_{it + 1} = \frac{{I_{it + 1}^{Q} }}{{Z_{it + 1} }} \times 100.$$

The productivity growth indices at regional level are calculated as the average indices of the LAs in each region. Means and standard deviations were calculated based on the conditional mean methods for each GOR.

### Data source and variable specification

#### Output data

As this paper measures productivity for care home services for older people, only one activity is measured—residential and nursing home services for older people (those who are 65 and over). Output is measured in Great Britain Pound (£). Data were drawn from PSSEX from National Adult Social Care Intelligence Service (NASCIS) 2010 to 2012.

As noted, the adjustment of quality is derived from the individual level analysis of the Adult Social Care Survey (ASCS) of 2010 to 2012. This survey collects data from service users on SCRQoL using the ASCOT indicator. The main variables for individual level need were also taken from the ASCS data: the scores of seven Activity of Daily Living (ADL) questions, one Instrumental ADL (IADL) question, two EQ-5D questions and self-assessed health. Table 1 lists the variables used to estimate the quality adjustment index.

**Table 1:** Variable specification for quality adjustment

	Variable specification	Data source
Dependent variables		
ASCOT (for service user above 65 residential and nursing care)	An average ASCOT score for each LA is used This score included eight items: Personal cleanliness and comfort Accommodation cleanliness and comfort Food and drink Safety Social participation and involvement Occupation Control over daily life Dignity	ASCS 2010, 2011, and 2012
Independent variables		
Service user health needs variables	Percentage of male service users EQ5D—pain and discomfort EQ5D—anxiety and depression IADL (instrumental ADL) Being able to deal with finances/paperwork ADL Being able to get in/out bed/chair by yourself Being able to feed yourself Being able to wash all over by yourself using bath or shower Being able to get dressed/undressed by yourself Being able to use WC/toilet by yourself Being able to wash face and hands by yourself Self-assessment health—good, average, and poor health	ASCS 2010, 2011, and 2012

#### Input data

We used (deflated) expenditure data to calculate our input growth index. Specifically, data for 2010 to 2012 for each local authority in England were used. Since we are interested in productivity with regard to publicly funded services, we use current expenditure (i.e., excluding capital charges) as the input. The deflator used in this analysis is the Personal Social Services (PSS) Pay and Prices Index [23]. The results do not change to any substantive degree when other expenditure metrics (i.e., net total expenditure) are used. For output and input data, we dropped LAs without full input and output information: Cheshire (North West), Derbyshire (East Midlands), Bedfordshire (Eastern), Nottinghamshire (East Midlands), Suffolk (Eastern), Milton Keynes (South East), Cornwall (South West), Slough (South East), Camden (London), Richmond-upon-Thames (London), Isles of Scilly (South West) and City of London (London).

## Results

### Quality adjustment

Using the ASCS data, the LA fixed-effect model (3) was estimated in Stata13. The regression results are given in Table 2.^2^ The results show that,* ceteris paribus*, needs (i.e., self-assessment health, ADL, EQ5D) are significantly associated with the ASCOT. Females are more likely to report higher ASCOT score compared to males. Non-white people are less likely to report higher ASCOT score compared to white people.

**Table 2:** Results from the fixed-effect model using individual level data

Variables	Coefficient	S.E.
Demographic characteristics		
Female	0.0084**	[0.0021]
Non-white	−0.0288**	[0.0067]
Health needs		
Good health	0.0431**	[0.0022]
Poor health	−0.0818**	[0.0035]
ADL count (ref = 3)		
0	0.0103*	[0.0045]
1	0.0074+	[0.0044]
2	0.0017	[0.0047]
4	−0.0029	[0.0048]
5	−0.0133**	[0.0043]
6	−0.0352**	[0.0042]
7	−0.0863**	[0.0042]
IADL (ref = 0)	0.0034	[0.0033]
EQ5D pain (ref = 2)		
EQ5D pain 1	0.0131**	[0.0021]
EQ5D pain 3	−0.0229**	[0.0044]
EQ5D anxiety (ref = 2)		
EQ5D anxiety 1	0.0548**	[0.0021]
EQ5D anxiety 3	−0.1133**	[0.0049]
Year 2011	0.0089+	[0.0047]
Year 2012	0.0094+	[0.0048]
Constant	0.8281**	[0.0062]
LA dummies	Yes	
Interaction: LA × year 2011	Yes	
Interaction: LA × year 2012	Yes	
*N*	23,522	
*R*-squared	0.2791	

** *p* < 0.01, * p < 0.05, + *p* < 0.1. Base year is 2010

**Fig. 1:**
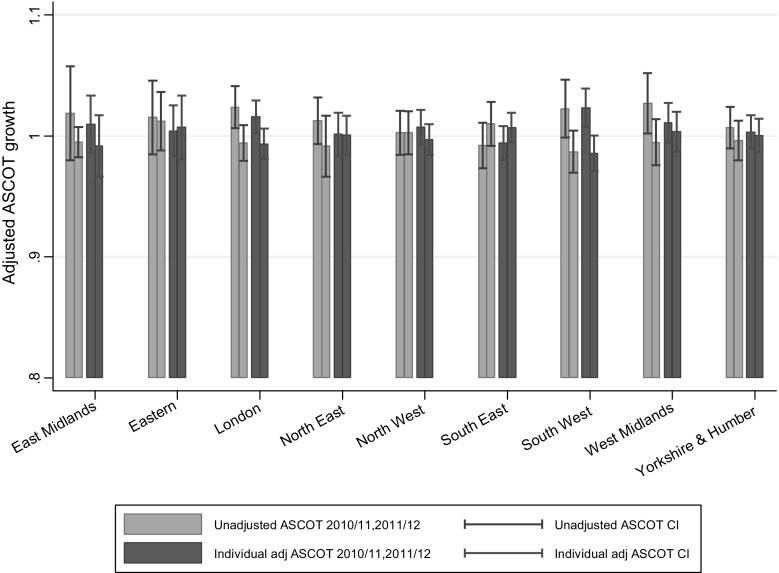
Unadjusted (raw) and individual-level adjusted quality of care using ASCOT by region by year

Figure 1 presents two quality adjustments. The first is the ratio of the year-on-year change in raw ASCOT score at LA level (for 2010–2011 and 2011–2012). The second calculates this ratio using the results of the individual-level regression method (the $\alpha_{it}$ value for each LA at each year) in (4). The latter, in other words, controls for individual need factors as discussed above. We estimate the quality ratios and their respective standard errors using delta method (nlcom command in Stata). The two adjustments are, respectively, denoted as the unadjusted ASCOT (raw ASCOT without any adjustment) and individual level data adjusted ASCOT in the figure.

In a number of cases, i.e., London 2010–2011 and the South West 2010–2011, the year-on-year change ratio was significantly different from one, suggesting that there was a significant change from one year to another. In terms of the different methods of adjustment, the individual level data approach appeared to show better precision (smaller confidence intervals (CI)) than using the unadjusted ASCOT approach.

### Output and input growth

Table 3 shows the output for older adult services from 2010 to 2012 by regions. From 2010 to 2011, output for all other regions increased except for East Midlands, South East, South West, and West Midlands. From 2011 to 2012, output for most regions increased except for the South West.

**Table 3:** Output for residential and nursing care for old adults by region by years (mean/SD) (£000’s)

Region		2010	2011	2012
East Midlands	7	36,224.43 (23,598.78)	36,153.7 (24,994.8)	36,708.14 (25,939.03)
Eastern	9	35,310.04 (33,264.19)	36,321.48 (33,144.73)	38,041.11 (35,177.43)
London	30	17,423.29 (4753.438)	18,523.58 (4658.394)	18,982.43 (4513.199)
North East	12	25,790.07 (16,872.81)	26,139.16 (16,885.09)	27,636.67 (16,852.17)
North West	23	27,989.87 (20,757.9)	28,743.82 (21,410.56)	30,722.26 (23,373.47)
South East	18	47,838.13 (46,499.57)	47,214.54 (47,273.75)	49,263.94 (47,116.8)
South West	14	36,181.21 (23,350.03)	35,545.64 (23,211.53)	34,784.14 (22,504.2)
West Midlands	14	32,778.69 (22,130.23)	32,638.1 (21,681.82)	35,128.64 (26,304.43)
Yorkshire and Humber	15	32,771.47 (15,000.52)	34,171.6 (14,456.89)	35,369.53 (16,523.05)
National average	142	30,742.29 (25,377.39)	31,179.31 (25,423.72)	32,416.37 (26,417.33)

Table 4 shows the regional cost-weighted output growth indices from 2010 to 2012. Three indices are presented: unadjusted, raw ASCOT adjusted, and individual level data adjusted output growth. The quality adjustment of output growth again produced somewhat different results from output changes without quality adjustment. For example, the quality-adjusted output growth index for London grew significantly between 2011 and 2012 because the ratio was significantly different from one (the lower bound of CI is larger than one), whereas the unadjusted output growth was not statistically significant from one. By contrast, in the East Midlands, the unadjusted change ratio between 2010 and 2011 was significantly greater than one but the (individual-level) adjusted ratio was not significantly different.

**Table 4:** Unadjusted and quality-adjusted output growth by region by year (mean/CI)

Region	Unadjusted output growth (mean/CI)		ASCOT adjusted output growth (mean/CI)		Indi level adjusted output growth (mean/CI)	
	2010–2011	2011–2012	2010–2011	2011–2012	2010–2011	2011–2012
East Midlands	1.039 (1.009, 1.069)	1.009 (0.971, 1.048)	1.058 (1.009, 1.107)	1.004 (0.962, 1.046)	1.05 (0.995, 1.106)	1.002 (0.962, 1.041)
Eastern	1.024 (0.998, 1.05)	1.02 (0.982, 1.058)	1.039 (1.002, 1.076)	1.034 (0.977, 1.09)	1.024 (0.991, 1.056)	1.033 (0.98, 1.086)
London	1.038 (1.001, 1.075)	1.034 (1.003, 1.065)	1.061 (1.023, 1.1)	1.027 (0.996, 1.059)	1.053 (1.017, 1.088)	1.027 (0.997, 1.057)
North East	1.007 (0.974, 1.04)	1.014 (0.98, 1.047)	1.02 (0.98, 1.06)	1.006 (0.956, 1.056)	1.009 (0.971, 1.047)	1.016 (0.97, 1.061)
North West	1.025 (0.991, 1.059)	0.998 (0.971, 1.025)	1.029 (0.983, 1.075)	1 (0.968, 1.033)	1.041 (0.999, 1.083)	0.996 (0.963, 1.029)
South East	1.02 (0.994, 1.047)	1 (0.954, 1.047)	1.012 (0.979, 1.045)	1.009 (0.968, 1.05)	1.014 (0.985, 1.042)	1.007 (0.962, 1.053)
South West	0.96 (0.906, 1.015)	1.018 (0.966, 1.07)	0.981 (0.927, 1.034)	1.003 (0.959, 1.048)	0.983 (0.927, 1.039)	1.002 (0.957, 1.047)
West Midlands	1.003 (0.965, 1.042)	1.038 (1.016, 1.06)	1.029 (0.991, 1.068)	1.032 (1.009, 1.055)	1.015 (0.973, 1.057)	1.042 (1.013, 1.072)
Yorkshire and Humber	1.034 (0.994, 1.074)	1.031 (1.008, 1.054)	1.041 (0.994, 1.089)	1.027 (0.996, 1.058)	1.038 (0.99, 1.087)	1.031 (1, 1.062)

Table 5 shows regional inputs in cash terms and real terms (PSS deflated) for older adult services from 2010 to 2012. Table 6 presents both un-deflated and PSS deflated input growth indices from 2010 to 2012. The results showed from 2010 to 2011, PSS deflated input for all other regions decreased except for South West. From 2011 to 2012, PSS deflated input for all other regions increased except for London and Yorkshire and Humber.

**Table 5:** Input for residential and nursing care for all adult and old adults based on net current expenditures by region by years (£000’s) (mean/S.D.)

	2010	2011		2012	
	Base year	Cash term	Real term	Cash term	Real term
East Midlands	23,864.71 (17,697.87)	22,836 (15,926.34)	23,066.67 (16,087.21)	22,271.29 (15,180.26)	23,199.26 (15,812.77)
Eastern	25,772.11 (24,176.89)	24,707.44 (24,030.68)	24,957.01 (24,273.42)	24,415.78 (23,910.01)	25,433.1 (24,906.26)
London	13,628.17 (3608.004)	13,166.37 (3253.526)	13,299.36 (3286.39)	12,184.57 (3499.294)	12,692.26 (3645.098)
North East	18,771.67 (12,223.57)	16,803.83 (10,352.03)	16,973.57 (10,456.59)	16,665.92 (9976.078)	17,360.33 (10,391.75)
North West	19,965.78 (15,439.1)	18,018.48 (13,120.35)	18,200.48 (13,252.88)	17,716.09 (12,796.73)	18,454.26 (13,329.93)
South East	31,965.61 (30,537.32)	29,562.17 (29,300.47)	29,860.77 (29,596.43)	30,692.5 (29,649.19)	31,971.35 (30,884.57)
South West	23,335.64 (15,150.85)	23,604.79 (15,911.61)	23,843.22 (16,072.33)	24,094.43 (15,981.89)	25,098.36 (16,647.81)
West Midlands	24,177.14 (19,246.24)	20,881.64 (13,996.79)	21,092.57 (14,138.17)	21,388.07 (14,162.74)	22,279.24 (14,752.86)
Yorkshire and Humber	22,512.6 (11,498.73)	21,231.73 (9915.843)	21,446.19 (10,016)	20,260.67 (10,415.47)	21,104.86 (10,849.45)
National average	21,623.73 (17,597.21)	20,187.93 (16,188.19)	20,391.85 (16,351.7)	20,012.46 (16,420.39)	20,846.31 (17,104.57)

**Table 6:** Input growth indices for residential and nursing care for older adult services (mean/S.D.)

Region	2010–2011		2011–2012	
	Net current expenditure growth	Net current expenditure growth (pss deflated)	Net current expenditure growth	Net current expenditure growth (pss deflated)
East Midlands	0.974 (0.111)	0.983 (0.112)	0.984 (0.098)	1.025 (0.102)
Eastern	0.957 (0.067)	0.967 (0.068)	0.977 (0.138)	1.018 (0.144)
London	0.98 (0.13)	0.989 (0.132)	0.923 (0.112)	0.961 (0.117)
North East	0.9 (0.085)	0.909 (0.086)	1.008 (0.114)	1.05 (0.119)
North West	0.936 (0.126)	0.945 (0.127)	0.987 (0.126)	1.028 (0.132)
South East	0.945 (0.173)	0.955 (0.175)	1.042 (0.096)	1.085 (0.1)
South West	1.003 (0.107)	1.013 (0.108)	1.027 (0.058)	1.07 (0.06)
West Midlands	0.897 (0.127)	0.906 (0.128)	1.033 (0.101)	1.076 (0.105)
Yorkshire and Humber	0.952 (0.087)	0.961 (0.088)	0.944 (0.086)	0.983 (0.09)
National average	0.951 (0.123)	0.961 (0.125)	0.985 (0.112)	1.026 (0.117)

### Productivity

Productivity growth index is the ratio of output growth divided by the ratio of input growth. Table 7 presents indices: without any quality adjustment; with ASCOT adjustment; and with the individual-level quality adjustment. We use net current expenditure (PSS deflated) to calculate input growth ratio. Using the quality-adjusted measures, productivity growth was positive between 2010 and 2011 for all regions except South West (where there was no significant change). Productivity change was negative for South East and South West (the lower bound of CI is smaller than one), positive for London (the upper bound of CI is smaller than one), and remained unchanged (CI contains one) for other regions from 2011 to 2012.

**Table 7:** Unadjusted and quality adjusted productivity growth by region by year (mean/CI)

Region	Unadjusted productivity growth (mean/CI)		ASCOT adjusted productivity growth (mean/CI)		Indi level adjusted productivity growth (mean/CI)	
	2010–2011	2011–2012	2010–2011	2011–2012	2010–2011	2011–2012
East Midlands	1.066 (0.988, 1.143)	0.994 (0.9, 1.088)	1.083 (1.018, 1.148)	0.989 (0.891, 1.088)	1.074 (1.01, 1.138)	0.987 (0.886, 1.089)
Eastern	1.063 (1.01, 1.116)	1.019 (0.925, 1.114)	1.08 (1.011, 1.15)	1.033 (0.929, 1.136)	1.062 (1.009, 1.115)	1.031 (0.933, 1.129)
London	1.063 (1.008, 1.117)	1.093 (1.028, 1.158)	1.088 (1.028, 1.148)	1.083 (1.027, 1.14)	1.08 (1.021, 1.139)	1.083 (1.027, 1.14)
North East	1.116 (1.052, 1.18)	0.977 (0.905, 1.049)	1.129 (1.067, 1.191)	0.97 (0.89, 1.049)	1.116 (1.063, 1.17)	0.98 (0.899, 1.061)
North West	1.102 (1.037, 1.167)	0.986 (0.928, 1.045)	1.104 (1.038, 1.17)	0.987 (0.932, 1.042)	1.117 (1.052, 1.182)	0.983 (0.927, 1.04)
South East	1.102 (1.009, 1.195)	0.926 (0.88, 0.971)	1.094 (0.996, 1.192)	0.934 (0.892, 0.975)	1.095 (1.001, 1.189)	0.933 (0.888, 0.977)
South West	0.953 (0.897, 1.009)	0.956 (0.894, 1.018)	0.972 (0.921, 1.023)	0.942 (0.888, 0.995)	0.975 (0.918, 1.032)	0.941 (0.886, 0.995)
West Midlands	1.134 (1.023, 1.245)	0.973 (0.92, 1.025)	1.161 (1.058, 1.263)	0.968 (0.912, 1.023)	1.144 (1.042, 1.245)	0.976 (0.924, 1.028)
Yorkshire and Humber	1.088 (1.004, 1.172)	1.057 (0.999, 1.116)	1.096 (1.006, 1.186)	1.053 (0.993, 1.113)	1.093 (1.001, 1.185)	1.058 (0.995, 1.12)

The pattern was slightly different when considering the unadjusted productivity growth indices. Unadjusted productivity was not significantly changed: between 2010 and 2011 in the East Midlands; and between 2011 and 2012 in the South West, in contrast to the adjusted results.

**Fig. 2:**
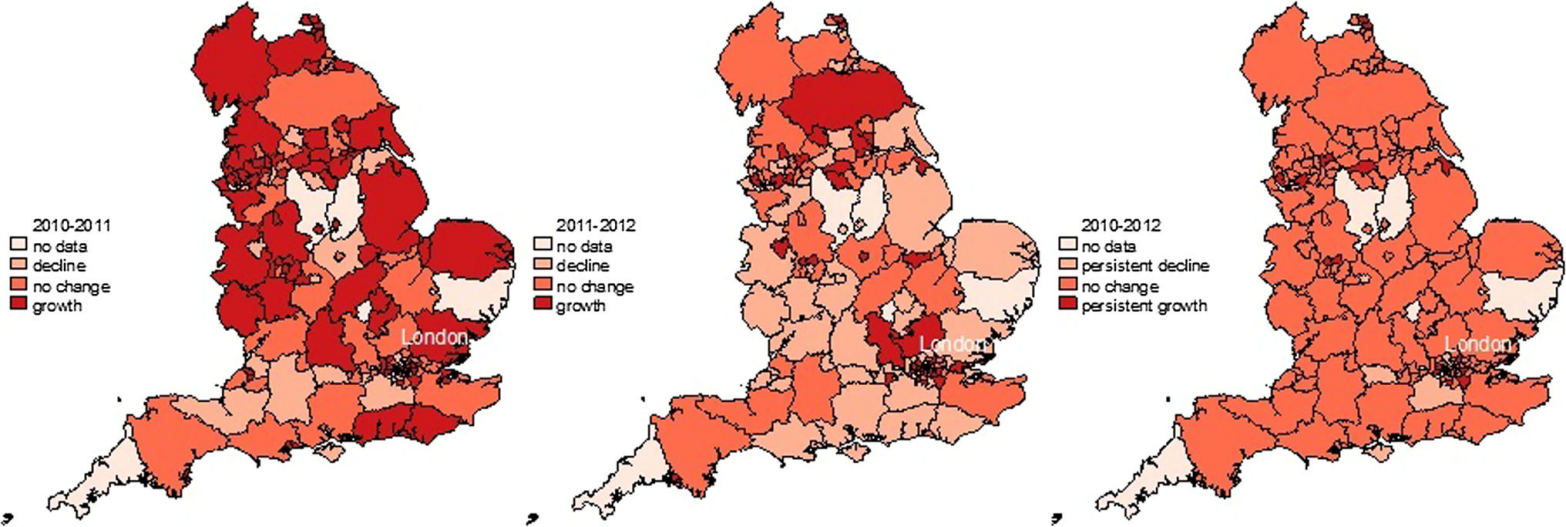
Productivity growth maps by LA by years. Note: the map for 2010–2012 represents consecutive changes for two periods of time

Figure 2 maps productivity growth at LA level across England. The first two maps show statistically significant changes in productivity over 2001–2011 and 2011–2012 period, respectively. The third map identifies regions that had persistent growth or persistent decline over both periods (i.e., consecutive periods of significant change in the same direction). The results were largely consistent with the regional level findings. A number of LAs, i.e., London and Buckinghamshire, demonstrated continuously positive productivity growth for the study period

### Robustness tests

We performed one set of robustness tests for the quality adjustment. Instead of using individual level data, we used data on the average ASCOT score aggregated to LA level from ASCS as the basis for quality adjustment. We allowed regional time effect in the equation to estimate directly the yearly regional quality change. We obtained similar results as the individual-level quality adjustment.

## Discussion and conclusions

The main aim of the care system, as clearly expressed in the 2014 Care Act, is to improve quality of life. As such, any assessment of productivity should be made in those terms. To date assessments of productivity in ASC have involved the measurement of outputs of services, not their impact on the outcome of recipients, per se. The reason is that doing the latter is challenging; not least, there are the technical problems of attribution and measurement to tackle. As a result, there are currently no data on the degree to which the use of specific services will improve the outcome of services users.

This paper, to our knowledge, is the first one to use service outputs data with quality adjustment. Moreover, the adjustment uses care-related quality of life (ASCOT) data, which is a good ‘operational’ measure of well-being. Attribution is addressed by controlling for observables but also specifying the adjustment in relative terms as a year-by-year index, and thereby limiting any attribution bias that is due to time-invariant factors.

Our aim in this regard was to adjust using a measure of the quality of care services. Because this is unobserved, we instead inferred service quality from data on social-care related quality of life (SCRQoL) of service users. The challenge is that SCRQoL is also a function of need and service intensity/input, as well as service quality. Our approach was to control as far as possible for need and implied service intensity changes using observed individual person need factors in an LA-level fixed effects regression analysis. Need in the population will vary, and this is a normal part of the way the care system operates. What is important in productivity terms is how much services improve quality of life of the person, not whether need has changed where this does not impact on how far services improve quality of life. The only exception to this principle is where given amounts of improvement in quality of life are valued more highly for high-need people than low-need people (i.e., where equity weights are applied). In this analysis, we assume changes are small enough, year-on-year, not to warrant equity considerations.

Our approach accounts for changes in need in as far as this affects changes in the impact of services to improve quality of life. For example, if need increased between periods, but observed SCRQoL did not change, then services must have got better at producing outcomes, i.e., productivity improved. But instead, if we observed that SCRQoL reduced by the amount expected for the change in need (as estimated), then service quality will not have improved; the care system would just be dealing with higher need people at the same level of effectiveness (quality), their productivity would not have changed. Although subject to practical limitations, as outlined below, the method does in theory differentiate between these two cases, accounting for quality of life and need simultaneously.

We have focused on the ‘outputs’ side of the productivity equation, arguing the need to make quality-of-life adjustments. Nonetheless, the method does accommodate both changes in inputs—see Eq. (6)—and (before-quality-adjustment) outputs as potentially impacting on productivity.

As a demonstration of the method, we estimated adjusted productivity ratios for the 3 years 2010 to 2012 for residential and nursing care among older people. Using quality-adjusted productivity growth measures, we found that the productivity growth of residential and nursing care for older people increased for most regions from 2010 to 2011, and remained unchanged for most regions from 2011 to 2012.

The methods used allow us to assess productivity change for individual LAs, which can be aggregated up to the regional level. As well as estimating national productivity change, the approach taken in this study allows us to compare year-on-year productivity changes by locality. By measuring productivity growth in different regions, we are able to identify underperforming regions, and demonstrate areas where potential savings can be made. However, we only have data for 3 years in this study. When, in future years, we have data to compare regions over a longer period, we will be able to assess differences in productivity trends between areas, and nationally.

There are many possible factors that influence productivity change, some of which are local issues and some national. The changing national policy context is certainly relevant. For instance, the allocation of additional budget from NHS to ASC in 2011 is considered as an important policy change, the amount of spending reported by local authorities may have changed accordingly. The analysis of overall productivity trends is not designed to associate observed changes to particular factors. Nonetheless, greater insight can be gained in this regard by comparing trends between areas.

In considering the implications of this analysis, we must also bear in mind potential limitations. As a basis for quality adjustment, we used the ASCOT score from the annual adult social care survey. Although this provides rich individual level data, a self-completion survey like the ASCS is restricted to people using LA services who are sufficiently free from impairment so as to be able to complete the questionnaire. Also, the survey only covers people currently in receipt of services, i.e., the eligible population. LAs control for eligibility, but the experiences of people below eligibility thresholds would not be taken into account. Although ASCS is a cross-sectional survey, it is possible that respondents may appear in more than one wave of the survey. We are not able to account for this in the regression model and, as a result, the standard errors may be under-estimated.

In terms of the inputs calculation, we are only able to use input data from current and total expenditures for councils in England. We cannot separate expenditures from different input sources, i.e., labor, intermediate and capital input. In order to calculate input growth, indirect measures of input growth was applied, which was a less ideal measure to use compared with the direct measures. In this analysis, we adjusted service quality by controlling for possible attribution problems using LA and individual level data. However, apart from these, there might be other factors associated with quality of services, which cannot be captured by the current methods. The analysis is based on 142 LAs that have full data, which represents a partial picture of the England region. Any generalizations of the study results should be made with caution. Our goal was primarily to demonstrate the method. As more data become available, the results will be more robust.

Despite the limitations, we have provided an approach to account for quality and need in assessing productivity. This approach is feasible with current data and it has a significant impact on the results compared with non-adjusted productivity metrics. Estimation of productivity trends, especially by region, will be valuable information for the development of policy in adult social care.

## Footnotes

1 We ran a regression controlling for the available regional level characteristics (such as number of population above 85, number of people receiving benefits, etc.) and found no statistically significant and close to zero in magnitude effect on the individual measure of SCRQoL, which implies $(\alpha_{2} \cong 0).$

2 LA-level and interaction effects are available from the authors.
